# Biomedical Image Analysis Special Applications in MRIs and CT Scans

**DOI:** 10.5195/jmla.2025.2104

**Published:** 2025-04-18

**Authors:** Evita Muthi'atul Maula, Vanela Chatrin Lekatompessy, Selfi Selfi, Renaldo Apriandi Kasa, Faza Atika An'umillah

**Affiliations:** 1 evitamaula25@gmail.com, Department of Physics, Faculty of Mathematics and Natural Science, Brawijaya University, Indonesia; 2 vanelachatrin@gmail.com, Department of Biology, Faculty of Mathematics and Natural Science, Brawijaya University, Indonesia; 3 selfisilfi@gmail.com, Department of Physics, Faculty of Mathematics and Natural Science, Brawijaya University, Indonesia; 4 randikasa20@gmail.com, Department of Physics, Faculty of Mathematics and Natural Science, Brawijaya University, Indonesia; 5 fazaatika24@gmail.com, Department of Physics, Faculty of Mathematics and Natural Science, Brawijaya University, Indonesia

Biomedical Image Analysis Special Applications in MRIs and CT Scans is a part of the Brain Informatics of Health (BIH) Book series. This book covers the fundamental theory of these techniques and their practical applications through various examples, presented in a straightforward manner without complex mathematics. The authors delve into key aspects of biomedical image analysis, including model formulation, architecture, basic steps, empirical analysis, and performance evaluation using statistical parameters to assess the effectiveness of the proposed models.

The series is a leading resource in brain informatics and computational brain studies, offering a comprehensive review of brain informatics and health topics. It also covers advanced and recent topics, serving as a platform for emerging subjects that are not yet suitable for textbooks.

The book is targeted at researchers in biomedical image analysis, computer science, and research organizations. It is ideal for individuals interested in applying soft computing techniques to biomedical image analysis, specifically for MRI and CT scans. It is also suitable for those looking to grasp the techniques used in medical image data processing without delving into overly complex mathematics.

The editors introduce the first chapter, “Introduction,” by highlighting the significance of medical image analysis, particularly MRI and CT scans, in diagnosing medical conditions. They discuss key image processing techniques like segmentation and clustering, and address the difficulties encountered in analyzing medical images. This chapter lays the foundation for the methodologies discussed in subsequent chapters.

Parkinson's disease is a progressive central nervous system disorder that affects motor and balance functions. While an MRI scan can help detect the disease early and predict symptom severity, it cannot definitively diagnose Parkinson's. Chapters 2, 3, and 4 explore different MRI analysis methods for Parkinson's disease. Chapter 2 introduces the Fuzzy Clustering Method, which aims to identify brain changes associated with Parkinson's by addressing uncertainty in MRI images. Experimental results demonstrate its effectiveness. Chapter 3 presents the Neutrosophic-Entropy Segmentation method, designed to detect subtle brain structure changes in Parkinson's patients using an entropy-based segmentation algorithm. The method's performance is evaluated and compared with other techniques. In Chapter 4, the Neutrosophic-Entropy Clustering Method is discussed, focusing on clustering MRI images to identify patterns related to Parkinson's disease. This approach groups pixels based on uncertainty levels, enhancing pattern detection. Chapter 5 introduces a segmentation method for brain tumor detection in MRI images using type-2 neutrosophic theory-based thresholding, improving accuracy in tumor identification. Lastly, a quantum clustering approach for CT image segmentation of COVID-19 patients is discussed in the final chapter, combining K-means clustering with a quantum optimization algorithm for efficient large-scale medical data processing, particularly in lung analysis for COVID-19 cases.

This book is outstanding and offers a wide range of benefits. Its clear structure facilitates easy navigation through the chapters, ensuring a smooth flow of discussion. The practical applications, such as MRI and CT scan analysis, help readers connect theory with real-world scenarios, enhancing comprehension. The book covers diverse approaches, including various algorithms like K-means, neutrosophic sets, and fuzzy information gain, providing a compre-hensive overview of medical image processing techniques. The focus on health issues underscores the significance of science in advancing disease diagnosis and treatment. The introduction of new algorithms reflects ongoing efforts to enhance efficiency and effectiveness in medical image clustering and segmentation. Researchers and practitioners seeking to deepen their understanding of these techniques will find this book to be a valuable reference. The systematic presentation of each chapter ensures that readers can easily grasp the information and apply it in relevant contexts.

